# Towards the creation of a flexible classification scheme for voluntarily reported transfusion and laboratory safety events

**DOI:** 10.1186/2041-1480-3-4

**Published:** 2012-05-18

**Authors:** Julie M Whitehurst, John Schroder, Dave Leonard, Monica M Horvath, Heidi Cozart, Jeffrey Ferranti

**Affiliations:** 1 Duke Health Technology Solutions, Duke University Health System, 2424 Erwin Road, Suite 1201, Durham, NC, 27705, USA; 2 Department of Pediatrics, Duke University School of Medicine, Durham, NC, USA

## Abstract

**Background:**

Transfusion and clinical laboratory services are high-volume activities involving complicated workflows across both ambulatory and inpatient environments. As a result, there are many opportunities for safety lapses, leading to patient harm and increased costs. Organizational techniques such as voluntary safety event reporting are commonly used to identify and prioritize risk areas across care settings. Creation of functional, standardized safety data structures that facilitate effective exploratory examination is therefore essential to drive quality improvement interventions. Unfortunately, voluntarily reported adverse event data can often be unstructured or ambiguously defined.

**Results:**

To address this problem, we sought to create a “best-of-breed” patient safety classification for data contained in the Duke University Health System Safety Reporting System (SRS). Our approach was to implement the internationally recognized World Health Organization International Classification for Patient Safety Framework, supplemented with additional data points relevant to our organization. Data selection and integration into the hierarchical framework is discussed, as well as placement of the classification into the SRS. We evaluated the impact of the new SRS classification on system usage through comparisons of monthly average report rates and completion times before and after implementation. Monthly average inpatient transfusion reports decreased from 102.1 ± 14.3 to 91.6 ± 11.2, with the proportion of transfusion reports in our system remaining consistent before and after implementation. Monthly average transfusion report rates in the outpatient and homecare environments were not significantly different. Significant increases in clinical lab report rates were present across inpatient and outpatient environments, with the proportion of lab reports increasing after implementation. Report completion times increased modestly but not significantly from a practical standpoint.

**Conclusions:**

A common safety vocabulary can facilitate integration of information from disparate systems and processes to permit meaningful measurement and interpretation of data to improve safety within and across organizations. Formation of a “best-of-breed” classification for voluntary reporting necessitates an internal examination of localized data needs and workflow in order to design a product that enables comprehensive data capture. A team of clinical, safety, and information technology experts is necessary to integrate the data structures into the reporting system. We have found that a “best-of-breed” patient safety classification provides a solid, extensible model for adverse event analysis, healthcare leader communication, and intervention identification.

## Background

Transfusion and clinical laboratory services are integral parts of inpatient and outpatient care that heavily affect outcomes and costs [1-3]. Laboratory data may influence up to 70% of important decisions made throughout a hospital stay, and up to 2.7 million blood product transfusions occur yearly in the United States [2,4]. Given the immense use of these services, it is inevitable that serious safety events arise. Preventable errors have been shown to cause patient suffering, permanent disability, and death [5,6]. As a result, blood transfusion and clinical laboratory safety have been priorities for both governmental and accrediting healthcare organizations. In the United States, the Food and Drug Administration Center for Biologics Evaluation and Research (CBER) inspects facilities according to specific quality standards [7] and the reporting of fatalities related to blood collection, transfusion, or medical device use is mandatory [8,9]. Accrediting agencies such as the College of American Pathologists (CAP) provide comprehensive safety standards [10], and The Joint Commission (TJC) prioritizes problems with incompatible blood transfusions, laboratory patient identification, and communication by designating them as National Patient Safety Goals [11]. Additionally, incompatible blood transfusions are specified as “serious reportable events” and “never events” by the Centers for Medicare and Medicaid Services (CMS) and the National Quality Forum (NQF), respectively [12,13].

Clearly, the healthcare community has great motivation to ensure safe practices in blood product and clinical laboratory environments. One of the most effective monitoring techniques has been to use adverse event and “near-miss” data to direct and measure quality improvement efforts [14]. Many healthcare sites have voluntary reporting systems that capture various event types in either paper or electronic forms [15,16]. Examination of data such as contributing factors, system failures, and outcomes may facilitate discovery of event trends that can be used to inform safety initiatives, broaden communication, and enhance care coordination [17].

Voluntarily reported events are often captured via free-text narratives, and formal data schemas for structured data are frequently lacking. These shortcomings are possibly due to an urgent demand for event reporting system development following estimates that 98,000 patients may die annually from medical errors in the United States alone, as well as the fact that proper terminology development requires substantial effort [17,18]. To accurately and consistently identify event trends, thorough examination of standardized, codified aggregate data and the capacity to drill into granular detail is greatly needed [17,19]. However, the addition of structure to data does not in itself solve analysis challenges if there are interpretation differences in definitions and terms across healthcare sites [17]. Thoughtful use of safety data may facilitate risk reduction, allow for benchmarking, spur predictive modeling, and satisfy regulatory reporting requirements. But if data are not properly created and collected, the attainment of these benefits may be substantially diminished [17].

In order to facilitate meaningful data exploration of transfusion and laboratory-related safety events across the Duke University Health System (DUHS), we sought selection of a standardized patient safety vocabulary that could be incorporated into our voluntary Safety Reporting System (SRS). A classification that serves as a foundation for such knowledge generation must consist of hierarchically arranged and defined data that allow comparisons across event types, patient populations, and disciplines.

Diverse, detailed safety classifications and taxonomies have previously been applied; however, many are restricted to a single safety event type or patient population, thus diminishing extensibility [20-22]. The World Health Organization Conceptual Framework for the International Classification for Patient Safety (ICPS) [23-25] is a data model containing defined, universal patient safety concepts that make global accumulation of meaningful safety information possible. Safety leaders have promoted and continue to develop the classification with a goal of assimilating safety information from disparate systems into a single common format for use [26]. Specific, labeled safety concepts of interest, all linked through semantic associations, are grouped into clinically meaningful classes, which yield plentiful data for exploration and analysis. Defined, high-level classes include incident type, incident characteristics, patient characteristics, contributing factors/hazards, patient outcomes, organizational outcomes, detection, mitigating factors, ameliorating actions, and actions taken to reduce risk. Subdivisions exist within each class, allowing for capture of more granular event details.

The ICPS is intended to be interoperable with existing classifications, allowing it to be customized to meet the unique data collection requirements of specific organizations and thus create a more comprehensive classification [24]. At DUHS, we recognized that creation of a “best-of-breed” safety classification for SRS-reported transfusion and laboratory-related events may drive standardized reporting that can be readily translated into actions to reduce risk. Our goal was to build and implement an organized data structure for voluntary reporting that would enable aggregate report creation and increase our capacity to benchmark. Therefore, we decided to use the ICPS framework as a basis for data structure within SRS.

The SRS is a Web-based, internally developed application created in 2002 that is used for voluntary patient safety reporting at the DUHS [27-29]. Using SRS, DUHS staff may report events anonymously in a non-punitive manner. SRS serves many DUHS locations, including one academic medical center, two community hospitals, numerous outpatient clinics, and a home care and hospice practice. Approximately 1600 events are submitted monthly across all sites, and all DUHS employees have access to report an event. Following submission, reports are directed to appropriate patient safety leaders and clinical managers (known as “reviewers”) who examine the reports and define immediate actions to remedy any adverse outcomes and lower risk of recurrence. SRS currently has approximately 9,400 reviewers across the health system. Data are aggregated and translated into action plans by numerous safety and quality committees. SRS is a confidential reporting system with medical peer review and quality assurance protections afforded under North Carolina law. These qualities promote a culture of safety [14] that enables SRS to serve as a driver of quality improvement across DUHS.

## Methods

### Safety classification creation and database design

Prior to this project, safety event descriptions, comments, or recommendations at DUHS were entered by both reporters and reviewers as free text within SRS. Codified data were minimal, with reports consisting of simple codified checklists allowing reporters to select various details (e.g., type of transfusion event), contributing factors (e.g., staff or environmental factors), injuries, and interventions that occurred. The few codified data points did not have agreed-upon definitions and therefore were subject to inconsistent usage. Codified data were used to form basic aggregate reports; however, reviewers in multiple departments maintained manually collected data outside of SRS to categorize data according to their own definitions and to respond to regulatory and accrediting agencies. Clinical laboratory reports were isolated within a generic “treatment and testing” pathway, which also included physical and respiratory therapy events. As a result, many clinical laboratory events were frequently reported through the “miscellaneous” event category, as reporters could not easily locate the correct placement. Consequently, SRS reviewers often had inadequate information and turned to patient chart review or staff discussion. Given these inefficiencies, experts requested a patient safety classification to assist quality improvement initiatives.

Design of the enhanced SRS patient portal began in 2009. A total of two safety analysts who serve as administrators of SRS, two experts in transfusion and clinical laboratory safety, and three front-line staff members worked to select data elements to build a safety classification that will allow for practical application of the ICPS framework. The team reviewed existing data sets and classification schemes to select data elements that were clinically applicable within our healthcare institutions. Concepts contained in the ICPS framework classes were reviewed, as well as data elements that are important to capture for internal and external reporting to organizations such as the U.S. Food and Drug Administration (FDA), College of American Pathologists (CAP), and AABB (previously the American Association of Blood Banks) [30]. In addition, other applicable safety event taxonomies were examined for data to incorporate, including the Medical Event Reporting System for Transfusion Medicine (MERS-TM) [31] and the relatively new Common Formats from the U.S. Agency for Healthcare Research and Quality (AHRQ) [32]. Inclusion of these data elements is important for comprehensive data capture, but also necessary for potential future automated submission of data to respective programs (e.g., submission of AHRQ Common Format data elements to a Patient Safety Organization). Internal data elements not contained in any specific data set or classification were also incorporated, as these were useful data that were collected before this project. Data elements are named using internal and organizational medical terminology, much of which is translatable to staff at other healthcare organizations. Data elements were then classified in hierarchies by the same project group, according to concept definitions from the ICPS framework. We used the ICPS high-level class structure as the model for our data, placing each data element in its applicable class and subdivisions. The classification schemes are available in Additional file 1. Broadly, the following ICPS concepts represent the highest level of hierarchy used: patient characteristics, incident characteristics, contributing factors, person reporting, and actions to reduce risk. Fifteen data elements populate 11 subclasses in the clinical labs classification scheme, and 129 data elements populate 37 subclasses in the transfusion classification scheme. Each scheme has a maximal depth of four tiers. For example, the transfusion incident data element “blood product sent for incorrect patient” is classified under the ICPS class of “blood product” incident type (tier 1), with further classification as a “wrong patient problem” (tier 2) according to the class subdivisions. This element is entered by the Reporter (entry source column; Additional file 1) and depends upon the Reporter having selected “yes” in response to whether the incident occurred in direct patient care (dependency column; Additional file 1). Similarly, a transfusion data element for “transfusion reaction” would have the same entry mode and dependency but would be classified as an “adverse effect” (tier 2) within the ICPS class of “blood product” incident type (tier 1). Group consensus was reached during data selection and classification. Formal reliability testing was not performed, as our goal was to practically apply the ICPS framework to SRS through creation of the best-of-breed classification. The hierarchical class structure enables quality leaders to apply descriptions consistently, drive data aggregation and analysis through business intelligence, and facilitate predictive modeling. The data model facilitates current data aggregation practices while allowing translatability through ICPS, therefore making the model easily adaptable to other healthcare sites.

**Figure 1:**
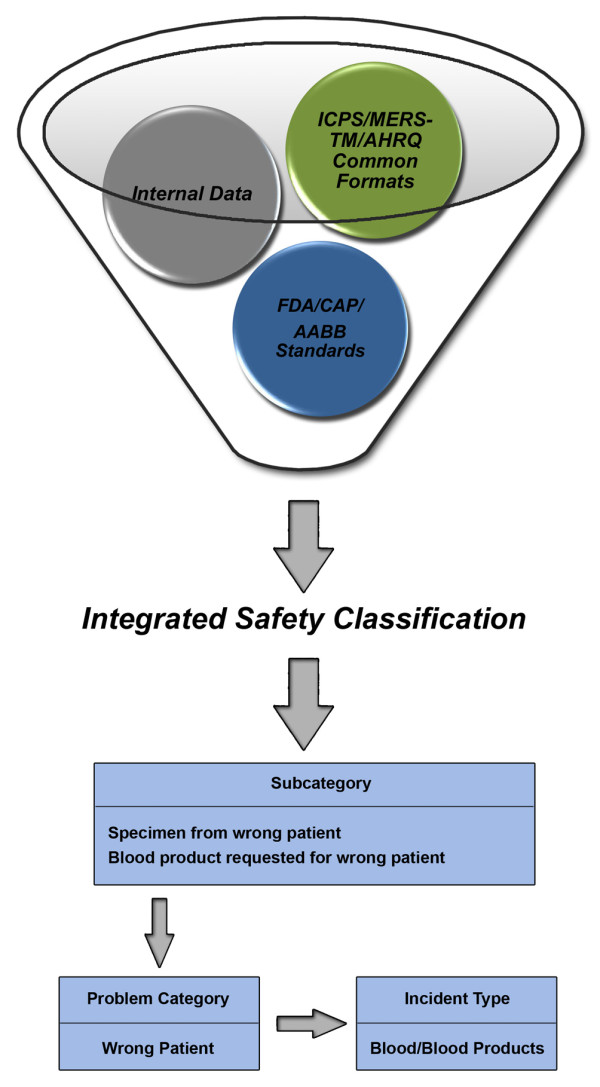
**Approach to “best-of-breed” safety classification creation.** Data points of interest from multiple sources including the ICPS, MERS-TM, AHRQ Common Formats, and standards from regulatory and accrediting agencies such as the FDA, CAP, and AABB were incorporated to create comprehensive transfusion and laboratory safety classifications. Questions for each indicator utilizing internal terminology were developed, while the data points themselves were mapped to appropriate classes within the ICPS framework in the SRS database schema. For example, patient identification related data are classified under “wrong patient”, which facilitates aggregate reporting for this category.

The SRS database schema was updated to accommodate the new safety classification, allowing us to transform the front-end reporter data into a standardized nomenclature on the back end. Each data point was appropriately categorized in the relational database to facilitate analysis among multiple levels of data aggregation (Figure 1). The classification is flexible, as additions and deletions can now easily be made to accommodate future changes that may occur within the ICPS framework.

### Front-end system design and implementation

Because entry of complex patient safety data may prove challenging to some reporters, attention was paid to designing a system that could accommodate users with limited healthcare experience and varying educational backgrounds and skill sets. Interface design is of utmost importance because efficiency can influence reporting, barriers to which may include complex questions, lack of time, and perceived absence of value [33].

**Figure 2:**
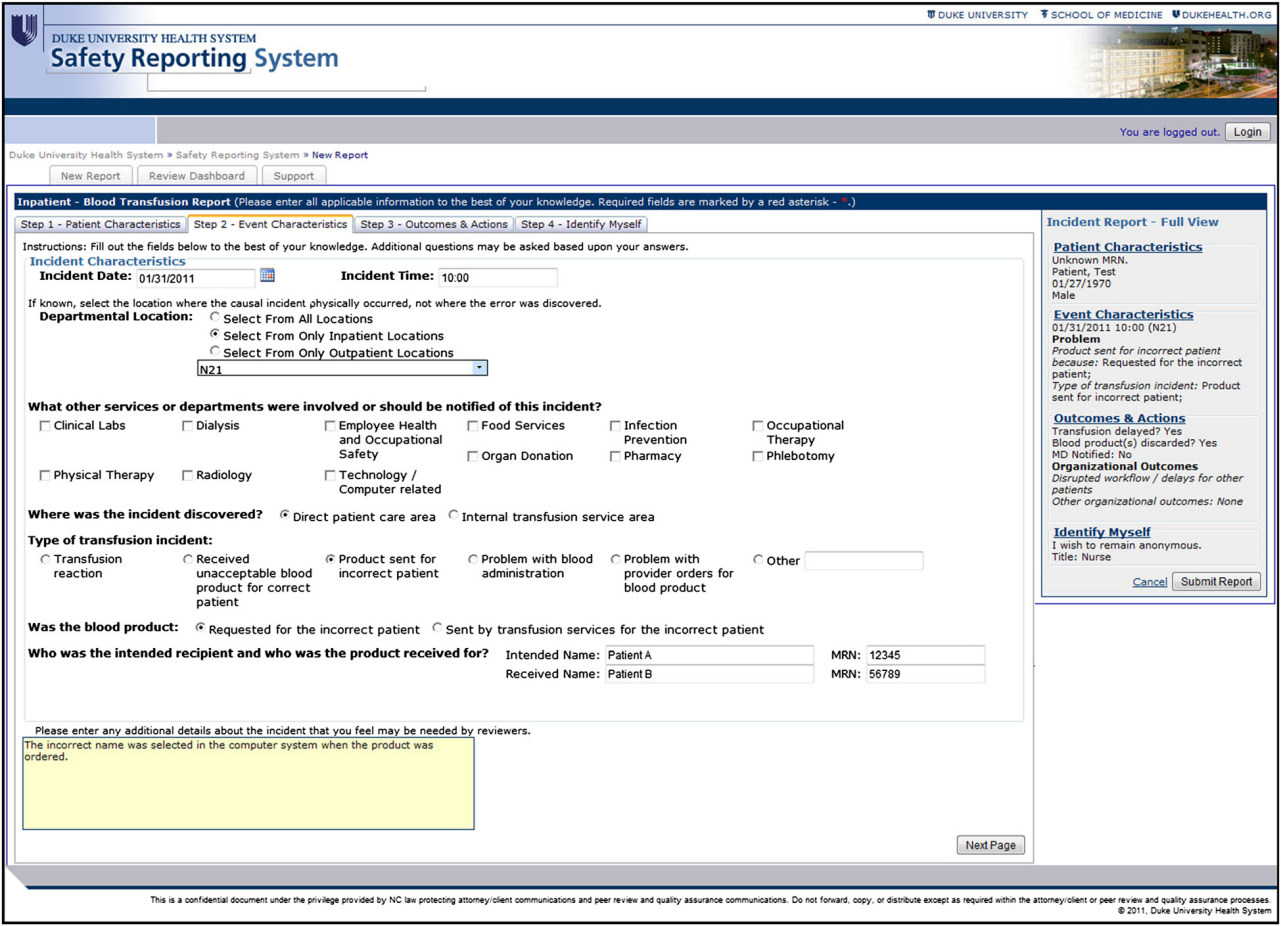
Blood transfusion safety reporting system reporter interface (abbreviated) post implementation of the integrated safety classification.

To address these barriers, we first created a standardized reporting form to enable consistent data collection across event types (Figure 2). Event data are collected in four steps driven by the safety classification content, including patient characteristics, incident characteristics, outcomes and actions, and reporter demographics. To address reporting time, a series of focused, codified questions with connected dependencies was built allowing select question-and-answer fields to appear or disappear according to previous answer choices, thus permitting reporters to complete a report without viewing every question.

By creating a series of succinct and logical safety event questions on the front end, we sought to maintain efficiency while accurately collecting event details. Use of familiar vocabulary present in daily staff workflow allowed us to overcome the problem of consistently educating reporters about definitions of ICPS concepts while maintaining common definitions and safety vocabulary on the back end. All codified answer fields are required to ensure comprehensive data collection. Because free text may be needed for uncodified information [34], a text box for event narratives was placed at the bottom of the webpage. Questions were limited to one webpage to lessen potential technical delays, and a side summary panel that automatically populates as a report is completed was created for comprehensive data views regardless of reporting step.

To promote usefulness of reports to staff, we enabled reporters to gain reviewer access to participate in the review process and directly view feedback, which we expect positively influences reporting rates and encourages a culture of safety [35]. In addition, the SRS reviewer interface was revised from a text-based format to a systematic data view, and editing capability was added to promote data accuracy. Reviewers may select codified actions to reduce risk and submit textual comments.

Prior to go-live, the new portal was evaluated during two test phases followed by functional and technical revisions. Front-line clinicians and organizational patient safety leaders performed testing and submitted feedback regarding content and efficiency. Comments were positive and only minor interface changes were suggested. Technical testing was performed by SRS analysts. To advertise these changes, health–system-wide training materials were produced for distribution. In addition to brief electronic modules specific to reporter and reviewer functionality, numerous informational training sessions were held across DUHS.

### Evaluation

Assessment of the effects of the new classification was essential to characterize the impact that implementation had on reporting, as the method by which voluntary information is collected may reduce reporting or influence data quality [33]. We estimated the impact of the redesigned SRS on usage pre- and post- classification implementation using monthly report rates and completion times (in minutes). Completion time is defined as the interval between the time that an individual first creates a new SRS report and the time that they click the “submit” button, indicating report completion as collected by the system log files. Data included in the “pre” period were events reported between the last major system release and classification implementation (April 1, 2008-February 9, 2010 and April 1, 2008-November 9, 2009 for transfusion and laboratory, respectively). Reports included in the “post” period included those reported after each implementation date until January 31, 2011.

Data were analyzed by patient environment (inpatient, outpatient, or homecare/hospice), with homecare/hospice being applicable only to the clinical laboratory category because transfusions are not performed within that environment. Chi-square and t-tests were used to compare categorical and continuous data, respectively. The Wilcoxon rank sum test was used to compare nonparametric data. All statistical analysis was performed using JMP Pro 9.0 (SAS Corporation, Cary, NC, USA).

## Results and discussion

The pre- and post- classification implementation statistics for transfusion and laboratory reports are displayed in Tables 1 and 2. The monthly average number of inpatient transfusion SRS reports was statistically different from post-implementation, decreasing from 102.1 ± 14.3 to 91.6 ± 11.2. This decrease should be interpreted with caution, as the proportion of transfusion reports during each period was not significantly different (8.6 vs. 7.9, p = 0.3238), so the monthly rate decrease may be due to seasonal reporting effects. It may be expected that the proportion of reported events not change, as blood product safety has been a high priority and greatly regulated area by the FDA [36].

**Table 1:** Transfusion report rates and time for report completion

**Transfusion average reports per month**	**Inpatient**	**Outpatient**
*Pre-period (22 months)*		
Average number (± standard deviation)	102.1 ± 14.3	11.5 ± 14.3
*Post-period (12 months)*		
Average number (± standard deviation)	91.6 ± 11.2	11.7 ± 2.5
P value	0.03*	0.9
**Minutes to submit a transfusion safety report**^†^	**Inpatient**	**Outpatient**
*Pre-period (22 months)*		
Median time in minutes (range)	6.13	7.72
	(1.4-58.65)	(1.65-48.55)
*Post-period (12 months)*		
Median time in minutes (range)	7.26	8.81
	(1.72-50.17)	(2.17-32.15)
P value	0.0001^††^	0.02^††^

Pre-period: April 1, 2008–February 9, 2010; Post-period: February 10, 2010–January 31, 2011.

*Significant difference based on *t*-test.

^†^Excludes outliers ≥ 60 minutes (0.6% of reports).

^††^Significant difference based on Wilcoxon rank-sum test.

**Table 2:** Laboratory report rates and time for report completion

**Laboratory average reports per month**	**Inpatient**	**Outpatient**	**Homecare/Hospice**
*Pre-period (19 months)*			
Average number (± standard deviation)	29.1 ± 7.5	18.5 ± 5.8	0.2 ± 0.4
*Post-period (15 months)*			
Average number (± standard deviation)	50.7 ± 9.6	41.5 ± 13.7	0.6 ± 0.6
P value	<0.0001^*^	<0.0001^*^	0.03
**Minutes to submit a laboratory safety report**^†^	**Inpatient**	**Outpatient**	**Homecare/Hospice**
*Pre-period (19 months)*			
Median time in minutes (range)	9.13	11.15	19.03
	(1.93-52.82)	(2.67-58.98)	(8.7-30.9)
*Post-period (15 months)*			
Median time in minutes (range)	10.27	11.12	11.9
	(1.87-56.73)	(2.32-56.93)	(5.37-17.63)
P value	<0.0001^††^	0.9149	0.1655

Pre-period: April 1, 2008–November 9, 2009; Post-period: November 10, 2009-January 31, 2011.

*Significant difference based on *t*-test.

^†^Excludes outliers ≥60 minutes (2.3% of reports).

^††^Significant difference based on Wilcoxon rank-sum test.

**Figure 3:**
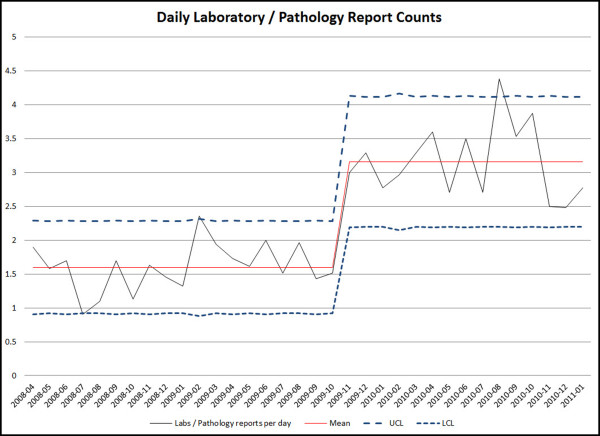
**Clinical laboratory report numbers before and following safety classification implementation.** A clear increase in the number of laboratory reports per day occurred after pathway implementation. “Pre” laboratory reports were manually classified from within the treatment and testing category for comparison to laboratory reports numbers after implementation. LCL: lower control limit; UCL: upper control limit.

In the outpatient and homecare environments, monthly rates were not significantly different, indicating that classification implementation did not affect the desire to report. For laboratory reports, analysis required manual review of reports within the SRS “treatment/testing” category to isolate true laboratory reports for the pre-study phase, given that a laboratory category did not exist prior to implementation. When these numbers are compared with those after implementation, significant increases in average monthly report rates exist for both the inpatient and outpatient environments (p <0.001). An obvious increase in the number of laboratory reports per day across all DUHS sites is displayed in Figure 3.

The homecare/hospice environment did not demonstrate a significant difference at this time, which may be due to a smaller number of reports overall when compared with other environments. We are continuing data collection to further compare pre- and post- report quantities in the future. It should also be noted that SRS interface changes, as well as enabling reporters to be involved in the report review process, may have produced a more favorable reporting culture, which could have increased reporting.

When examining reporting times, we deemed an increase in report completion times of 5 minutes or more to be a significant barrier to reporting. Some increases in reporting times should be anticipated, given that more data are now collected from reporters. We discovered a wide range in report completion times because many front-line reporters multitask while filling out the reports. When analyzing the data, report completion time outliers (≥60 minutes) were excluded to give a more accurate representation of the true time to finish a report, which included 0.6% and 2.3% of transfusion and laboratory data, respectively. Median transfusion reporting times were significantly different in both inpatient and outpatient care environments; however, the increases were well below our threshold of 5 minutes (Tables 1 and 2). A significant change was also found in the inpatient environment for laboratory reports; however, the median increase again was well below 5 minutes. Laboratory report completion times for outpatient and homecare environments decreased.

It is recognized that the current absence of a common patient safety vocabulary is a significant hindrance to communication of safety lessons learned across the continuum of healthcare [37]. A common vocabulary structure for large volumes of voluntarily reported data can empower front-line clinicians and safety leaders with the ability to easily understand where problems may reside within their institutions, and it can facilitate comparisons on regional and national levels when extended across systems [17]. A large, translatable data pool adds power to analyses performed to assist us in understanding each other’s areas for improvement. The ICPS provides a uniform classification for integrating safety information across disparate systems and processes into a common format for global use [24].

Given the high use of voluntary reporting systems in healthcare today, it is logical to apply the ICPS to these systems in order to generate a high yield of patient safety data [15]. In order to promote success, development of a classification reflective of internal workflow and practices should be considered, as those that do not utilize current local practices may result in user dissatisfaction and decreased use of the reporting system. Local clinical and safety experts as well as those involved in regulatory/accreditation processes should be involved in the “best-of-breed” design process to ensure that comprehensive content is present. Following classification development, a team dedicated to design, technical development, and deployment is necessary for successful product launch.

Our evaluation of the integration of the “best-of-breed” classification into the DUHS SRS indicates product acceptance, as we saw only minimal increases in reporting times below 5 minutes, no decreases in transfusion event report numbers, and increases in clinical laboratory event report numbers. We expected some increases in report completion time due to the larger volume of data being collected. Formal system usability testing was not conducted due to funding barriers. This ideally should be performed to isolate additional problematic areas for classification implementation. Usability of voluntary reporting systems is an infrequently studied topic within the medical literature, and the acquisition of knowledge in this area would be beneficial to all who intend to modify their voluntary reporting systems. We intend to apply the ICPS “best-of-breed” methodology to other patient safety event categories within SRS.

### Limitations

This work has several limitations. First, reporters are asked to classify the incident information themselves, meaning that different reporters may potentially reconcile incident characteristics in different ways. The magnitude of this issue could be assessed by measuring the inter-rater reliability of a group of randomly selected reporters against a test dataset of SRS reports, which is out of scope of this study. However, because reports may come from any employee of the health system, it is impractical to ensure that all reporters classify information in a consistent manner. The reviewer role is critical in this instance to promote data accuracy; however, this issue is endemic to any voluntary reporting system and not unique to this study. Second, the evaluation framework is heavily confounded by the fact that the SRS reporting terminology changed concomitant with a redesigned user interface. Given this, it would be impossible to know whether changes in reporting rates or completion times were due to interface usability, terminology clarity, or both.

## Conclusion

Standardization of patient safety data can enhance adverse event reporting, aggregation, and analysis. Our overarching goal is to attain a standardized safety data structure to produce accessible data for query and local and international comparisons. Meaningful safety data allow for the development of evidence-based actions that can be prospectively applied to patient care processes and provide a means for intervention validation. Patient safety leaders should concentrate on classification development and integration into their reporting structures to help ensure application of meaningful data to improve patient safety. Our “best-of-breed” classification can serve as a model to other organizations seeking to integrate standardized data collection into their operational functions. As we continue work on this project, we will consider funding avenues to reach out to the greater patient safety community to improve the terminology.

## Availability of supporting data

The WHO International Classification for Patient Safety (ICPS) development platform can be explored at http://www.who.int/patientsafety/implementation/taxonomy/development_site/en/index.html.

The Medical Event Reporting System for Transfusion Medicine (MERS-TM) glossary may be accessed at http://psnet.ahrq.gov/resource.aspx?resourceID=1066.

The most current version of the Common Formats for reporting safety events from the U.S. Agency for Healthcare Research and Quality (AHRQ) can be accessed here http://www.pso.ahrq.gov/formats/commonfmt.htm.

Additional file 1 describes the detailed best of breed classification schemes built from the ICPS, MERS-TM, and Common Formats systems.

## Abbreviations

AHRQ, Agency for Healthcare Research and Quality; CAP, College of American Pathologists; CBER, Center for Biologics Evaluation and Research; CMS, Centers for Medicare and Medicaid Services; DUHS, Duke University Health System; ICPS, International Classification for Patient Safety; MERS-TM, Medical Event Reporting System for Transfusion Medicine; NQF, National Quality Forum; SRS, Safety Reporting System; TJC, The Joint Commission.

## Competing interests

The authors declare that they have no competing interests.

## Authors’ contributions

JW, JS, DL, HC, and JF participated in project design and implementation. JW and MH contributed to writing of the manuscript. MH conducted data analysis. All authors approved the manuscript.

## Authors’ information

JW is a clinical pharmacist with the Health Analytics and IT Patient Safety group for DHTS (Durham, North Carolina). She designs and deploys technology solutions for safety and quality applications across Duke Medicine. She also provides project management services for the team.

JS is a clinical analyst with the Maestro Care (Epic) inpatient deployment team for DHTS (Durham, North Carolina). He obtained his bachelor of nursing degree from Duke University. Previously, he designed and provided support for the Safety Reporting System and its business intelligence platform.

DL is a senior IT analyst with the Maestro Care (Epic) deployment team for DHTS (Durham, North Carolina). He was the original architect and programmer of the Safety Reporting System and continues contribution to its development.

MH is the team lead for Health Intelligence and Research Services for DHTS (Durham, North Carolina). She serves as the statistics and analytics expert for evaluating the impact of health IT interventions.

HC is the senior director of the Maestro Care (Epic) inpatient deployment team for Duke Medicine (Durham, North Carolina). She has been involved in informatics initiatives focused on patient safety and outcomes research, software design, and major clinical system integration and deployment. Her research interests include measuring the impact of IT interventions on patient care processes and outcomes.

JF is the chief medical information officer for Duke Medicine (Durham, North Carolina). He is responsible for leading a team charged with the visioning, strategic planning, and effective adoption of integrated technology and information solutions that enable high quality clinical care, research, and education. He is an assistant professor of pediatrics and informatics, and holds a master’s degree in medical informatics.

## Funding

The authors and those named in the acknowledgements had no funding sources that supported this work.

## Supplementary Material

Additional file 1 **This file shows the classification scheme as a separate attachment given its size.** Here all data points can be viewed hierarchically by ICPS framework high-level class and subdivisions (column 1), data elements according to local language (column 2), the entry source of each data point (column 3), as well as data point dependencies that were used when implementing the classification into our safety reporting system (column 4). Click here for file
