# Supplementary material for: Towards the creation of a flexible classification scheme for voluntarily reported transfusion and laboratory safety events
Source: J Biomed Semantics. 2012 May 18;3:4. doi: 10.1186/2041-1480-3-4 (PMC3431246; doi:10.1186/2041-1480-3-4)
Supplement: Additional file 1 — This file shows the classification scheme as a separate attachment given its size. Here all data points can be viewed hierarchically by ICPS framework high-level class and subdivisions (column 1), data elements according to local language (column 2), the entry source of each data point (column 3), as well as data point dependencies that were used when implementing the classification into our safety reporting system (column 4). [file 2041-1480-3-4-S1.doc]

**Legend: Data elements (represented as DUHS values) are categorized in ICPS framework classes. The source of data input into SRS is noted, as some data are collected from reporters and reviewers of events and some are automatically populated from the clinical data repository. Data point dependencies are noted, illustrating which questions are dependent on ones previously asked during the report submission process. This promotes efficiency, as reporters are not required to answer every question when submitting reports.**

Table 1. Clinical Labs Classification Scheme

| ***ICPS Data Classification*** | ***Duke University Health System Value*** | ***Entry Source*** | ***Dependency (Data Point Dependent Upon)*** |
| --- | --- | --- | --- |
| Patient Characteristics    ⭭ Patient Demographics | Name | Reporter/  Automated | No |
| Patient Characteristics    ⭭ Patient Demographics | Medical record number | Reporter/  Automated | No |
| Patient Characteristics    ⭭ Patient Demographics | Age | Automated | No |
| Patient Characteristics    ⭭ Patient Demographics | Date of birth | Automated | No |
| Patient Characteristics    ⭭ Patient Demographics | Gender | Automated | No |
| Contributing Factors   ⭭ Incident Characteristics   ⭭ People Involved   ⭭ Healthcare Professional | RN, LPN, NCA, PT, MD | Reporter | No |
| Person Reporting    ⭭ Healthcare Professional | Reporter Title:  Advanced Practice Nurse  Manager/Director  Nurse  Nursing/Medical Assistant  Performance Improvement  Pharmacist  Physician  Physician Assistant  Practice Manager/HCA  Radiology  Rehab Services  Respiratory  Risk Manager  Social Worker  Transfusion Services | Reporter | No |
| Person Reporting    ⭭ Healthcare Worker | Reporter Title:  HUC/CSC  Pharmacy Technician | Reporter | No |
| Actions to Reduce Risk   ⭭ Patient Factors   ⭭ Provision of Patient Education/Training | Provide/Enhance Patient Education | Reviewer | No |
| Actions to Reduce Risk   ⭭ Staff Factors   ⭭ Training | Provide/Enhance Staff Education and/or Training | Reviewer | No |
| Actions to Reduce Risk   ⭭ Staff Factors    ⭭ Communication | Improve Staff Communication/Handoff Care | Reviewer | No |
| Actions to Reduce Risk   ⭭ Staff Factors   ⭭ Adequate Staff Numbers/Quality | Staffing Adjustment | Reviewer | No |
| Actions to Reduce Risk   ⭭ Agent/Equipment Factors   ⭭ Provision of Equipment | Equipment Repair or Replacement  Apply Safety Equipment | Reviewer | No |
| Actions to Reduce Risk   ⭭ Organizational/Environmental Factors    ⭭ Matching Physical Environment to Needs | Physical Environment Hazard Reduction | Reviewer | No |
| Actions to Reduce Risk   ⭭ Organizational/Environmental Factors   ⭭ Performing Risk Assessment/Root Cause Analyses | RCA | Reviewer | No |

Table 2. Transfusion Classification Scheme

| ***ICPS Data Classification*** | ***Duke University Health System Value*** | ***Entry Source*** | ***Dependency (Data Point Dependent Upon)*** |
| --- | --- | --- | --- |
| Patient Characteristics    ⭭ Patient Demographics | Name | Reporter/ Automated | No |
| Patient Characteristics    ⭭ Patient Demographics | Medical record number | Reporter/ Automated | No |
| Patient Characteristics    ⭭ Patient Demographics | Date of birth | Automated | No |
| Patient Characteristics    ⭭ Patient Demographics | Age | Automated | No |
| Patient Characteristics    ⭭ Patient Demographics | Gender | Automated | No |
| Incident Characteristics | Discovered in:  Direct patient care area  Internal transfusion services | Reporter | No |
| Problem    ⭭ Adverse Effect | Transfusion Reaction | Reporter | Yes  (direct patient care area) |
| Incident | Unacceptable blood product for correct patient | Reporter | Yes  (direct patient care area) |
| Problem    ⭭ Wrong patient | Product sent for incorrect patient | Reporter | Yes  (direct patient care area) |
| Incident | Problem with blood administration | Reporter | Yes  (direct patient care area) |
| Use Process   ⭭ Prescribing | Problem with provider orders for blood product | Reporter | Yes  (direct patient care area) |
| Blood/Blood Product Involved   ⭭ Cellular Products | Packed Red Blood Cells  Platelets | Reporter | Yes (transfusion reaction)  Yes (blood product unacceptable) |
| Blood/Blood Product Involved   ⭭ Albumin/Plasma Protein | Plasma/FFP | Reporter | Yes (transfusion reaction)  Yes (blood product unacceptable) |
| Blood/Blood Product Involved   ⭭ Clotting Factors | Cryoprecipitate | Reporter | Yes (transfusion reaction)  Yes (blood product |
| Staff Factors   ⭭ Cognitive Factors | Patient premedicated  Yes or No | Reporter | Yes (transfusion reaction) |
| Staff Factors   ⭭ Cognitive Factors | IDs of patient and blood product match | Reporter | Yes (transfusion reaction) |
| Patient Factors   ⭭ Pathophysiologic disease related factors | Patient fully catheterized:  Yes or No | Reporter | Yes (transfusion reaction) |
| Use Process   ⭭ Presentation/Packaging | Why product unacceptable:  Leaking bag  Product clotted | Reporter | Yes (received unacceptable blood product) |
| Use process   ⭭ Prescribing | Why product unacceptable: Patient’s transfusion modifications not met | Reporter | Yes (received unacceptable blood product) |
| Problem   ⭭ Wrong Product | Why product unacceptable: Incorrect blood product for correct patient | Reporter | Yes (received unacceptable blood product) |
| Staff Factors   ⭭ Cognitive Factors | Incorrect blood bank armband number | Reporter | Yes (received unacceptable blood product) |
| Staff Factors   ⭭ Cognitive Factors | Patient receive blood product: Yes or No | Reporter | Yes (received unacceptable blood product) |
| Staff Factors   ⭭ Cognitive Factors | Blood Product: Returned or Discarded | Reporter | Yes (received unacceptable blood product)  Yes (requested for incorrect patient) |
| Use process   ⭭ Prescribing | Modifications/Restrictions indicated on CPOE order form:  Yes or No | Reporter | Yes (received unacceptable blood product) |
| Staff Factors   ⭭ Cognitive Factors | Why product did not meet modifications:  Not irradiated  Not washed  Not volume reduced  Not HLA matched  Not CMV negative | Reporter | Yes (received unacceptable blood product) |
| Staff Factors   ⭭ Cognitive Factors | What was done with the blood product: Transfused, returned, discarded | Reporter | Yes (received unacceptable blood product)  Yes (requested for incorrect patient) |
| Problem   ⭭ Wrong patient | Product sent for incorrect patient:  Requested for incorrect patient  Sent by transfusion services for incorrect patient | Reporter | Yes (Product sent for incorrect patient) |
| Use Process   ⭭ Delivery | Blood administration problem:  Delay receiving blood product at bedside | Reporter | Yes (Blood administration problem) |
| Use Process   ⭭ Administration | Blood administration problem:  Delay in starting transfusion | Reporter | Yes (Blood administration problem) |
| Use Process   ⭭ Administration | Blood administration problem:  Transfusion lasting > 4 hours | Reporter | Yes (Blood administration problem) |
| Use Process   ⭭ Administration | Blood administration problem:  Administered with incorrect solution | Reporter | Yes (Blood administration problem) |
| Work/Environment Factors   ⭭ Equipment | Blood administration problem:  Equipment/supply issue | Reporter | Yes (Blood administration problem) |
| Work Environment Factors   ⭭ Equipment | Blood product receipt delayed because:  Pneumatic tube system problem | Reporter | Yes (Blood administration problem) |
| Use Process   ⭭ Preparation/Dispensing | Blood product receipt delayed because:  Product not ready | Reporter | Yes (Blood administration problem) |
| Staff Factors   ⭭ Communication Factors | Blood product receipt delayed because:  Failure to communicate product arrival on direct patient care unit | Reporter | Yes (Blood administration problem) |
| Patient Factors   ⭭ Pathophysiologic/Disease related factors | Transfusion lasted > 4 hours because:  IV access problem  Patient condition change | Reporter | Yes (Blood administration problem) |
| Patient Factors    | Transfusion delayed because:  Patient not on direct care unit | Reporter | Yes (Blood administration problem) |
| Organizational Factors   ⭭ Resources/Workload    | Transfusion delayed because:  Staffing issues/workload | Reporter | Yes (Blood administration problem) |
| Staff Factors   ⭭ Cognitive Factors    | Transfusion delayed because:  Delay in administering premedications | Reporter | Yes (Blood administration problem) |
| Patient Factors   ⭭ Pathophysiologic/Disease related factors | Transfusion delayed because:  IV access problem  Patient condition change | Reporter | Yes (Blood administration problem) |
| Use Process    ⭭ Prescribing | Why premedications delayed:  Premedication orders not placed | Reporter | Yes (Blood administration problem) |
| Use Process   ⭭ Preparation/Dispensing | Why premedications delayed:  Delay in obtaining premedications | Reporter | Yes (Blood administration problem) |
| Work Environment Factors   ⭭ Equipment | Problem with equipment:  Pump problem  Administration set problem | Reporter | Yes (Blood administration problem) |
| Staff factors   ⭭ Cognitive factors | Problem:  Failure to transfuse when order present  Transfusion with no order  Transfusion of blood product other than what was ordered to be transfused | Reporter | Yes (Problem with provider orders for blood product) |
| Problem   ⭭ Wrong Patient | Problem:  Provider completed order to prepare blood product for wrong patient | Reporter | Yes (Problem with provider orders for blood product) |
| Staff factors   ⭭ Cognitive factors | Problem with provider orders for blood product:  Patient transferred from another area  Order written prior to shift change  Order written on provider order from but not entered in CPOE | Reporter | Yes (Problem with provider orders for blood product-failure to transfuse when order present) |
| Staff factors   ⭭ Cognitive factors | Problem:  Acted on verbal order without written confirmation  Blood products ordered for preparation but no order to transfuse patient | Reporter | Yes (Problem with provider orders for blood product-transfusion with no order) |
| Problem   ⭭ Wrong patient | Problem:  Pick up slip for wrong patient | Reporter | Yes (Problem with provider orders for blood product-transfusion of blood product other than what was ordered to be transfused) |
| Staff factors   ⭭ Cognitive factors | Order to transfuse:  Yes or No | Reporter | Yes (order to prepare blood product for wrong patient) |
| Staff factors   ⭭ Cognitive factors | Product delivered to bedside:  Yes or No | Reporter | Yes (order to prepare blood product for wrong patient) |
| Staff factors   ⭭ Cognitive factors | Blood transfused:  Yes or No | Reporter | Yes (order to prepare blood product for wrong patient) |
| Incident | Type of incident:  Mishandled blood product | Reporter | Yes (discovered transfusion services) |
| Problem   ⭭  Wrong dispensing label/instruction | Type of incident:  Sample labeling problem | Reporter | Yes (discovered transfusion services) |
| Use Process   ⭭  Prescribing | Type of incident:  Incorrect blood component ordering | Reporter | Yes (discovered transfusion services) |
| Incident | Type of incident:  Blood component request (pick-up slip) problem | Reporter | Yes (discovered transfusion services) |
| Blood/Blood Product Involved   ⭭ Cellular Products | Packed Red Blood Cells  Platelets | Reporter | Yes (transfusion reaction)  Yes (blood product unacceptable) |
| Blood/Blood Product Involved   ⭭ Albumin/Plasma Protein | Plasma/FFP | Reporter | Yes (transfusion reaction)  Yes (blood product unacceptable) |
| Blood/Blood Product Involved   ⭭ Clotting Factors | Cryoprecipitate | Reporter | Yes (transfusion reaction)  Yes (blood product |
| Use Process   ⭭ Storage | Blood product:  Returned > 30 minutes after dispense (RBC or thawed plasma returned warm) | Reporter | Yes (mishandled blood product) |
| Use Process   ⭭ Delivery | Blood product:  Expired before pick-up | Reporter | Yes (mishandled blood product) |
| Use Process   ⭭ Storage | Blood product:  Stored improperly in cooler | Reporter | Yes (mishandled blood product) |
| Work environment factors   ⭭ Equipment | Blood product:  Lost in pneumatic tube | Reporter | Yes (mishandled blood product) |
| Staff Factors   ⭭ Cognitive Factor | Blood product:  Spiked but not transfused | Reporter | Yes (mishandled blood product) |
| Use Process   ⭭ Prescribing | Blood product returned because:  Requested more products than lines to transfuse | Reporter | Yes (mishandled blood product, return >30 min after dispense) |
| Problem   ⭭ Wrong patient | Blood product returned because:  Requested on wrong patient | Reporter | Yes (mishandled blood product, return >30 min after dispense) |
| Use Process   ⭭ Administration | Blood product returned because:  Patient had IV access problem | Reporter | Yes (mishandled blood product, return >30 min after dispense) |
| Use Process   ⭭ Delivery | Blood product returned because:  Sent to incorrect location | Reporter | Yes (mishandled blood product, return >30 min after dispense) |
| Use Process   ⭭ Prescribing | Blood product returned because:  Provider changed order | Reporter | Yes (mishandled blood product, return >30 min after dispense) |
| Use Process   ⭭ Administration | Blood product returned because:  Patient not available for transfusion | Reporter | Yes (mishandled blood product, return >30 min after dispense) |
| Staff Factors   ⭭ Cognitive Factors | Blood product returned because:  Transfusion not started within 30 minutes | Reporter | Yes (mishandled blood product, return >30 min after dispense) |
| Staff Factors   ⭭ Cognitive Factors | Blood product returned because:  Premedications not given | Reporter | Yes (mishandled blood product, return >30 min after dispense) |
| Work environment factors   ⭭ Equipment | Sent to incorrect location because:  Pneumatic tube system dysfunction | Reporter | Yes (mishandled blood product, return >30 min after dispense, sent to incorrect location) OR (mishandled blood product, lost in pneumatic tube) |
| Staff Factors   ⭭ Cognitive Factors | Sent to incorrect location because:  Wrong location entered by transfusion staff  Wrong location number on pick-up slip | Reporter | Yes (mishandled blood product, return >30 min after dispense, sent to incorrect location) OR (mishandled blood product, lost in pneumatic tube) |
| Patient Factors | Reason for delay:  Patient refused transfusion | Reporter | Yes (mishandled blood product, return >30 min after dispense, transfusion not started within 30 min) |
| Staff Factors   ⭭ Cognitive Factors | Reason for delay:  Nurse not notified of arrival of blood product | Reporter | Yes (mishandled blood product, return >30 min after dispense, transfusion not started within 30 min) |
| Patient Factors  ⭭  Pathophysiologic/Disease related factors | Reason for delay:  Patient coded | Reporter | Yes (mishandled blood product, return >30 min after dispense, transfusion not started within 30 min) |
| Use Process   ⭭ Prescribing | Reason premedications not given:  Order not found | Reporter | Yes (mishandled blood product, return >30 min after dispense, premedications not given) |
| Use Process   ⭭ Prescribing | Reason premedications not given:  Order not written in a timely fashion | Reporter | Yes (mishandled blood product, return >30 min after dispense, premedications not given) |
| Use Process   ⭭ Delivery | Reason premedications not given:  Delay in obtaining premedications | Reporter | Yes (mishandled blood product, return >30 min after dispense, premedications not given) |
| Use Process   ⭭ Storage | Event involved:  Cryoprecipitate returned in cooler  Platelets returned in cooler  RBCs on top of ice  Plasma on top of ice  Ice not in cooler  Cooler out > 24 hours | Reporter | Yes (mishandled blood product, stored improperly in cooler) |
| Problem    ⭭ Wrong dispensing label/Instruction | Problem with label:  Missing or incorrect date/time and/or initials  Name and/or MRN problem  Unlabeled sample  Paperwork/sample mismatch | Reporter | Yes (sample labeling problem) |
| Staff Factors | Problem with label:  Wrong blood in tube | Reporter | Yes (sample labeling problem) |
| People Involved   ⭭ Healthcare Professional | Who collected sample:  Phlebotomist  RN  MD  Resident  Medical student  Anesthesiologist | Reporter | Yes (sample label problem) |
| Staff Factors   ⭭ Cognitive Factors | Label not attached to sample tube?  Yes or No | Reporter | Yes (sample label problem, missing or incorrect date/time and/or initials) |
| Problem    ⭭ Wrong dispensing label/Instruction | Name or MRN Problem:  Incomplete name/MRN on label  Incorrect patient name/MRN on label  Omitted patient name/MRN on label | Reporter | Yes (sample label problem, name and/or MRN problem) |
| Work/Environment factors   ⭭ Equipment | Why name and/or MRN was incomplete:  Equipment problem | Reporter | Yes (sample label problem, name and/or MRN problem, incomplete name/MRN on label) |
| Staff Factors | Why name and/or MRN was incomplete:  Human Error | Reporter | Yes (sample label problem, name and/or MRN problem, incomplete name/MRN on label) |
| Problem    ⭭ Wrong dispensing label/Instruction | Labels in bag:  Yes or No | Reporter | Yes (sample label problem, unlabeled sample) |
| Staff Factors   ⭭ Cognitive Factors | Paperwork in bag:  Yes or No | Reporter | Yes (sample label problem, unlabeled sample) |
| Detection   ⭭ Process   ⭭ Error Recognition | Mislabeling discovered by:  Vital label in bag different from sample label  Historical type and sample type are different (historical type is wrong) | Reporter | Yes (sample label problem, wrong blood in tube) |
| Problem    ⭭ Wrong patient | Ordering problem:  Orders sent on wrong patient | Reporter | Yes (Incorrect blood component ordering) |
| Use Process   ⭭ Prescribing | Ordering problem:  Wrong product ordered to be prepared | Reporter | Yes (Incorrect blood component ordering) |
| Blood/Blood Product Involved   ⭭ Cellular Products | Packed Red Blood Cells  Platelets | Reporter | Yes (incorrect blood component ordering, wrong product ordered to be prepared)  Yes (blood component request problem, requested wrong product on patient)  Yes (blood component request problem, product other than what was requested issued) |
| Blood/Blood Product Involved   ⭭ Albumin/Plasma Protein | Plasma/FFP | Reporter | Yes (incorrect blood component ordering, wrong product ordered to be prepared)  Yes (blood component request problem, requested wrong product on patient)  Yes (blood component request problem, product other than what was requested issued) |
| Blood/Blood Product Involved   ⭭ Clotting Factors | Cryoprecipitate | Reporter | Yes (incorrect blood component ordering, wrong product ordered to be prepared)  Yes (blood component request problem, requested wrong product on patient)  Yes (blood component request problem, product other than what was requested issued) |
| Use Process   ⭭ Prescribing | Blood component request (pick-up slip) problem:  Requested wrong product on patient | Reporter | Yes (blood component request problem) |
| Problem    ⭭ Wrong patient | Blood component request (pick-up slip) problem:  Requested product for wrong patient | Reporter | Yes (blood component request problem) |
| Use Process   ⭭ Preparation/Dispensing | Blood component request (pick-up slip) problem:  Product other than what was requested issued | Reporter | Yes (blood component request problem) |
| Use Process   ⭭ Preparation/Dispensing | Blood component request (pick-up slip) problem:  Product for another patient was issued instead of the product for the right patient on whom the pick-up slip was received | Reporter | Yes (blood component request problem) |
| Use Process   ⭭ Prescribing | Blood component request (pick-up slip) problem:  Never received orders for product preparation | Reporter | Yes (blood component request problem) |
| Use Process   ⭭ Delivery | Blood component request (pick-up slip) problem:  Sent to incorrect patient location | Reporter | Yes (blood component request problem) |
| Work/Environment Factors    ⭭ Equipment | Why sent to incorrect location:  Pneumatic tube system malfunction | Reporter | Yes (blood component request problem, sent to incorrect location) |
| Staff Factors    ⭭ Cognitive Factors | Why sent to incorrect location:  Incorrect location on slip  Wrong location entered by technician | Reporter | Yes (blood component request problem, sent to incorrect location) |
| Patient Outcomes   ⭭ Type of Harm   ⭭ Pathophysiology   ⭭ Diseases of the blood & blood forming organs/disorders involving immune mechanisms | Patient exhibit symptoms of transfusion reaction:  Yes or No | Reporter | Yes (transfusion reaction incident) |
| Patient Outcomes   ⭭ Type of Harm   ⭭ Pathophysiology   ⭭ Diseases of the blood & blood forming organs/disorders involving immune mechanisms | Transfusion symptoms:  1) Elevated temperature > 1 degree C  2) Chills  3) Increased pulse (tachycardia)  4) Hives (urticaria)  5) Itching (pruritis)  6) Flushing  7) Hypotension  8) Increase in blood pressure  9) Dyspnea/tachypnea  10) Wheezing/stridor  11) Anaphylaxis  12) Failure to clot  13) Dark or red urine  14) Decreased urine output  15) Petechiae  16) Jaundice  17) Muscle aching  18) Acute or flank pain | Reporter | Yes (transfusion reaction incident) |
| Patient Factors  ⭭  Pathophysiologic/Disease related factors | Possible non-transfusion causes:  Yes or No | Reporter | Yes (transfusion reaction incident & exhibit symptoms) |
| Patient outcomes | Transfusion delayed:  Yes or No | Reporter | No |
| Organizational Outcomes   ⭭ Increase in required resource allocation for patient   ⭭ Additional treatment/tests    | Sample recollected:  Yes or No | Reporter | Yes |
| Organizational Outcomes   ⭭ Increase in required resource allocation for patient   ⭭ Additional treatment/tests | New product prepared:  Yes or No | Reporter | Yes |
| Organizational outcomes | Blood product discarded:  Yes or No | Reporter | No |
| Organizational Outcomes   ⭭ Increase in required resource allocation for patient | Resource allocation increased: Yes or No | Reporter | No |
| Organizational Outcomes   ⭭ Increased patient length of stay | Increased patient length of stay | Reporter | Yes (resource allocation increased) |
| Organizational Outcomes   ⭭ Patient admission to a special care area | Patient admission to a special care area | Reporter | Yes (resource allocation increased) |
| Organizational Outcomes   ⭭ Additional treatment or tests | Additional treatment or tests | Reporter | Yes (resource allocation increased) |
| Organizational Outcomes   ⭭ Disrupted workflow/delays for other patients | Disrupted workflow/delays for other patients | Reporter | Yes (resource allocation increased) |
| Organizational Outcomes   ⭭ Additional staff required | Additional staff required | Reporter | Yes (resource allocation increased) |
| Organizational Outcomes   ⭭ Additional equipment required | Additional equipment required | Reporter | Yes (resource allocation increased) |
| Organizational Outcomes | Other outcomes:  Formal complaint  Damaged reputation  Media attention  Legal ramifications | Reporter | No |
| Person Reporting    ⭭ Healthcare Professional | Reporter Title:  Advanced Practice Nurse  Manager/Director  Nurse  Nursing/Medical Assistant  Performance Improvement  Pharmacist  Physician  Physician Assistant  Practice Manager/HCA  Radiology  Rehab Services  Respiratory  Risk Manager  Social Worker  Transfusion Services | Reporter | No |
| Person Reporting    ⭭ Healthcare Worker | Reporter Title:  HUC/CSC  Pharmacy Technician | Reporter | No |
| Actions to Reduce Risk    ⭭ Staff Factors | Provide/Enhance staff education and/or training | Reviewer | No |
| Actions to Reduce Risk    ⭭ Staff Factors | Revise protocols | Reviewer | No |
| Actions to Reduce Risk    ⭭ Staff Factors | Improve staff communication/handoff care | Reviewer | No |
| Actions to Reduce Risk    ⭭ Staff Factors | Teamwork needed | Reviewer | No |
| Actions to Reduce Risk    ⭭ Staff Factors | Staffing adjustment | Reviewer | No |
| Actions to Reduce Risk    ⭭ Staff Factors | Increase procedure monitoring | Reviewer | No |
| Actions to Reduce Risk    ⭭ Agent/Equipment Factors | Equipment repair or replacement | Reviewer | No |
| Actions to Reduce Risk    ⭭ Organizational/Environmental Factors | RCA | Reviewer | No |
